# Homocystinuria: A rare condition presenting as stroke and megaloblastic anemia

**DOI:** 10.4103/1817-1745.76110

**Published:** 2010

**Authors:** Parveen Bhardwaj, Ravi Sharma, Minoo Sharma

**Affiliations:** Department of Pediatrics, Indira Gandhi Medical College, Shimla, Himachal Pradesh, India; 1 Department of Physiology, Indira Gandhi Medical College, Shimla, Himachal Pradesh, India

**Keywords:** Homocystinuria, megaloblastic anemia, stroke

## Abstract

Homocystinuria is an inborn error of amino acid metabolism in which homocystine accumulates in the blood and produces a slowly evolving clinical syndrome. We are presenting a case of a 4-year-old female child who presented to us with stroke and also had megaloblastic anemia. She was diagnosed as having homocystinuria type-1, and she responded to treatment.

## Introduction

The normal pathway of catabolism of methionine produces cystine, with homocysteine as a pivotal intermediate. Most homocysteine is normally remethylated to methionine. This methionine-sparing reaction is catalyzed by the enzyme methione synthase, which requires a metabolite of folic acid (5-methyltetrahydrofolate) as a methyl donor and a metabolite of vitamin B12 (methylcobalamin) as a cofactor. Further conversion of homocysteine to cystathionine requires a pyridoxal phosphate-dependent enzyme, cystathionine ß synthase, deficiency of which results in accumulation of homocysteine and reconversion of homocysteine to methionine.[1] Homocystinuria type-1, due to the deficiency of cystathionine ß synthase, is the most common inborn error of methionine metabolism. It is characterized by developmental delay, ectopia lentis, progressive mental retardation, skeleton abnormalities resembling Marfan syndrome and thromboembolic episodes. Diagnosis is usually made after 3 years of age, when most of the patients present with subluxation of the lens. Homocystinuria can also occur due to defect in methylcobalamin formation, i.e. homocystinuria type-II, characterized by the triad of megaloblastic anemia, homocystinuria and hypomethionemia. Deficiency of the enzyme methyltetrahydrofolate reductase results in homocystinuria type-III, characterized by homocystinemia, homocystinuria and low-low normal levels of methionine.[1,2] We encountered a patient who presented to us with stroke, had megaloblastic anemia and ectopia lentis. We made the diagnosis of homocystinuria type-I and instituted specific treatment.

## Case Report

A 4 year old female child presented to us with complains of weakness of the right half of the body. According to the mother of the child, 5 days back, the child had passed urine and stools in the bed and was crying excessively. When the mother tried to make the child stand up, she noticed that the child was not moving her right upper and right lower limbs. There were no H/O fever, rash, abnormal body movements, altered sensorium and pus discharge from the ear, no H/O bluish discoloration, breathlessness, syncopal attacks and palpitation, no H/O joint pains, petechial/purpuric/ecchymotic spots over the body and no H/O head/neck/oral trauma, no family H/O bleeding diathesis or cerebrovascular accidents. The child was immunized for age and her developmental and dietary histories were normal. The child was born to a nonconsangunious marriage and there was no history suggestive of homocystinuria in the family.

On examination, the child had fair complexion with light brown and woolly hair. Pallor was present. Rest of the general physical examination was normal. No marfanoid features, no neurocutaneous stigmata and no sternal tenderness were observed. Anthropometry was normal. There were no signs of any vitamin deficiency. Her vitals were stable.

Central nervous system-wise, the child was conscious, cooperative and well oriented. No cranial nerve palsy was present. There were no meningeal signs and no abnormal movements of the limbs. Muscle bulk was normal bilaterally. Tone was increased in the right upper and right lower limbs. Deep tendon reflexes were brisk in the right upper and right lower limbs. Power was 2/5 in the right upper and 3/5 in the right lower limb. Plantars were downgoing bilaterally. No cerebellar signs were present. The skull and spine were normal. Chest, cardiovascular system, abdomen and musculoskeletal system were normal. Eye examination showed bilateral inferonasal ectopia lentis. Investigations revealed a haemoglobin of 8 gm% and a normal total and differential leucocyte count. The platelet count was 140 × 10^3^/cmm. The reticulocyte count was 2.6% and Erythrocte sedimentation rate was 22mm in the 1^st^ hour. Renal function test, Liver function test, serum electrolytes and blood sugar were normal.

The peripheral smear showed anisopoikilocytosis, macroovalocytes, macrocytes, tear drop cells and polychromatic cells with coarse basophilic stippling. The mean corpuscular volume was 136 fL, the mean corpuscular hemoglobin was 36 pg, the mean corpuscular hemoglobin concentration was 30 g/dl and the bone marrow was suggestive of megaloblastic changes.

Magnetic resonance imaging of the brain showed moderate-sized hyperintensities in the left centrum semiovale and subtle hyperintensities in the bilateral caudate nuclei.

Sodium nitroprusside test in the urine was positive. The serum homocysteine level was 256.91 *μ*mol/L (normal range, 4.60–12.44). The serum vitamin B12 level was 310 pg/ml (normal range, 200–800 pg/ml). The fasting plasma methionine level was increased to 134.34 *μ*mol/L (normal range, 0–90 *μ*mol/L). Based on these investigations, we made the diagnosis of homocystinuria type-I and the patient was started on oral pyridoxine (400 mg/day) and oral folic acid (5 mg/day). Physiotherapy was also started. The child was also put on a low-protein, low-methionine diet. The child responded to treatment and started walking on day 5 of the treatment. The power was still 4/5 in the right upper limb at the time of discharge. Genetic counselling of the family was carried out. On follow-up after 3 months of treatment, the power was normal in the limbs, hemoglobin was 14 gm%, mean corpuscular volume decreased to 88.7 fL, mean corpuscular haemoglobin was 28.6 pg and mean corpuscular haemoglobin concentration was 33.68 g/dL. The platelet count increased to 180 × 10^3^/*μ*L. The serum homocysteine level was 52 *μ*mol/L (normal range, 4.60–12.44).

## Discussion

Homocystinuria is an autosomal recessively inherited defect in the transsulfuration pathway (type-I) or methylation pathway (types II and III). The internationally reported incidence of homocystinuria varies between 1 in 50,000 and 1 in 200,000.[3] Normally, homocysteine is an intracellular intermediate and is not detectable in plasma or urine. However, when the reconversion of homocysteine to methionine or cysteine is blocked, it accumulates extracellularly and results in homocystinuria.[4]

Infants with this disorder are normal at birth. Clinical manifestations during infancy are nonspecific, and may include failure to thrive and developmental delay. The diagnosis is usually made when subluxation of the ocular lens occurs. This causes severe myopia and iridiodonesis. Progressive mental retardation is common. Affected individuals with homocystinuria manifest skeletal abnormalities resembling those of Marfan syndrome. Children usually have fair complexions, blue eyes and a peculiar malar flush. Thromboembolic episodes involving both large and small vessels, especially those of the brain, are common and may occur at any age.

Classically, megaloblastic anemia has been known to be associated with homocystinuria type-II.[1] Not many cases of megaloblastic anemia have been seen with homocystinuria type-I. The mechanism of megaloblastic anemia seen in homocystinuria type-I is the development of folate deficiency due to excessive consumption of methyltetrahydrofolate in the methylation of homocysteine to form methionine.[4–6] Thrombocytopenia seen in our patient can be attributed to folate deficiency, which improved subsequently on treatment with folic acid.[7] The megaloblastic anemia seen in homocystinuria type-II responds to treatment with vitamin B12. However, treatment in type-I requires pyridoxine, which acts as a coenzyme for the enzyme cystathionine synthase and its administration results in greater binding of the enzyme with the substrate by a simple mass action.[2] Treatment with high doses of pyridoxine causes dramatic improvement in patients who are responsive to therapy (40%), as seen in our patient. The need for dietary restriction and its extent is controversial in patients with vitamin B6-responsive form. Treatment with Betaine has produced clinical improvement in patients who are unresponsive to pyridoxine therapy.[1] Ectopia lentis is specifically seen with type-I homocystinuria only.[8] Thromboembolic episodes involving both large and small vessels, especially those of the brain, are common in type-I homocystinuria and can occur at any age.[1] The risk for vascular disease is graded with respect to the level of homocysteine. However, no threshold abnormal value is accepted widely. Several factors have been suggested as the possible cause of accelerated vascular disease. These include - endothelial cell damage, smooth muscle cell proliferation, lipid abnormalities, upregulation of pro-thrombotic factors and downregulation of antithrombotic factors or endothelial-derived nitric oxide.[3]

Thus, we conclude that homocystinuria must be kept in mind in the differential diagnosis of pediatric stroke, looking for other clinical manifestations like ectopia lentis and fair complexion, light hair and presence of megaloblastic anemia. All patients with homocystinuria treated with pyridoxine should receive folate supplementation. The treatment of homocystinuria assumes greater significance because institution of specific treatment can prevent progression of this disease and associated complications.
